# Cytotoxicity and Microbicidal Activity of Commonly Used Organic Solvents: A Comparative Study and Application to a Standardized Extract from *Vaccinium macrocarpon*

**DOI:** 10.3390/toxics9050092

**Published:** 2021-04-21

**Authors:** Yana Ilieva, Lyudmila Dimitrova, Maya Margaritova Zaharieva, Mila Kaleva, Petko Alov, Ivanka Tsakovska, Tania Pencheva, Ivanka Pencheva-El Tibi, Hristo Najdenski, Ilza Pajeva

**Affiliations:** 1 Department of Infectious Microbiology, The Stephan Angeloff Institute of Microbiology, Bulgarian Academy of Sciences, 1113 Sofia, Bulgaria; illievayana@gmail.com (Y.I.); lus22@abv.bg (L.D.); zaharieva26@yahoo.com (M.M.Z.); milakalevavet@abv.bg (M.K.); hnajdenski@abv.bg (H.N.); 2 Department of QSAR and Molecular Modelling, Institute of Biophysics and Biomedical Engineering, Bulgarian Academy of Sciences, 1113 Sofia, Bulgaria; petko@biophys.bas.bg (P.A.); itsakovska@biomed.bas.bg (I.T.); tania.pencheva@biomed.bas.bg (T.P.); 3 Department of Pharmaceutical Chemistry, Faculty of Pharmacy, Medical University—Sofia, 1000 Sofia, Bulgaria; ivatibi@gmail.com

**Keywords:** organic solvents, in vitro cytotoxicity, cell lines, microbicidal effect, proanthocyanidins

## Abstract

The cytotoxicity and microbicidal capacity of seven organic solvents commonly applied for studying plant extracts and bioactive compounds were systematically investigated based on international standards. Four cell lines of normal (CCL-1, HaCaT) or tumor (A-375, A-431) tissue origin, seven bacterial and one fungal strain were used. The impact of the least toxic solvents in the determination of in vitro cytotoxicity was evaluated using a standardized extract from *Vaccinium macrocarpon* containing 54.2% *v/v* proanthocyanidins (CystiCran^®^). The solvents ethanol, methoxyethanol and polyethylene glycol were the least cytotoxic to all cell lines, with a maximum tolerated concentration (MTC) between 1 and 2% *v/v*. Ethanol, methanol and polyethylene glycol were mostly suitable for antimicrobial susceptibility testing, with minimum inhibitory concentrations (MICs) ≥ 25% *v/v*. The MTC values of the solvents dimethyl sulfoxide, dimethoxyethane and dimethylformamide varied from 0.03% to 1.09% *v/v*. The MICs of dimethyl sulfoxide, methoxyethanol and dimethoxyethane were in the range of 3.125–25% *v/v*. The cytotoxic effects of CystiCran^®^ on eukaryotic cell lines were directly proportional to the superimposed effect of the solvents used. The results of this study can be useful for selecting the appropriate solvents for in vitro estimation of the cytotoxic and growth inhibitory effects of bioactive molecules in eukaryotic and prokaryotic cells.

## 1. Introduction

Cytotoxicity testing and evaluation of antimicrobial activity, following robust international standards [1,2,3], are among the most widespread approaches for screening important biological properties of drugs, plant extracts or microbial-derived products, their bioactive constituents and newly synthesized compounds. They often run in parallel because every testing for antimicrobial susceptibility requires evaluation of the in vitro cytotoxicity as well. Each test procedure starts with the successful delivery of the tested material into the bioassay system, which, to a great extent, determines the result of the experiment. Achieving the right concentrations and even distribution of the substances throughout the volume of the bioassay system depends on their full dissolution into an appropriate solvent. The latter should not be toxic itself, and its physicochemical and biological properties should not interfere with the measurements of interest. Numerous organic solvents have been thoroughly studied during the last decades for their toxicity on various cell lines and bacterial species to be applied in cytotoxicity and microbicidal assays [4,5,6,7,8,9,10,11]. Despite all published data, the constantly increasing number of new cell lines and their different sensitivity to commonly used solvents depending on the tissue of origin require a constant update of the specific in vitro cytotoxicity of the solvents used. In addition, the information about the solvent tolerance to pathogenic bacteria does not sufficiently cover the broad spectrum of bacterial strains and solvents used for antimicrobial susceptibility testing (AST), which points out the necessity of expanding the available data.

Aiming to fill the gap of information about cytotoxicity and microbicidal effects of solvents on non-tumorigenic and tumorigenic cell lines, test bacterial or fungal strains, recommended by the international standards for toxicity testing and AST, we focused our study on the effects of the following seven widely used organic solvents: dimethoxyethane (DME), 2-methoxyethanol (MEtOH), dimethyl sulfoxide (DMSO), polyethylene glycol (PEG-400), dimethylformamide (DMF), ethanol (EtOH) and methanol (MeOH). These solvents are mostly applied for the dissolution of polar and nonpolar compounds or extracts in studies evaluating antineoplastic, antimicrobial and cytotoxic activities.

DMSO, EtOH and PEG have been tested on a large panel of cell lines (mostly tumorigenic) and microorganisms [5,6,7,8,10,11,12,13,14]. Standard protocols recommend DMSO for nonpolar, poorly water-soluble substances [15], as it is a universal amphiphilic solvent for drug delivery with no effect on drug binding [10]. However, only a maximum 0.5% DMSO volume fraction only is acceptable for most cell culture test systems, and greater concentrations are cytotoxic for many cell lines, with few exceptions, such as lymphocytes [2,16]. Furthermore, there is evidence that low concentrations of DMSO are highly relevant for interpreting cellular parameters evaluated in cell assays other than the determination of basic in vitro cytotoxicity. For 0.25–1%, DMSO affected immunomodulatory effects, such as production of interleukin (IL)-6 or reactive oxygen species production induced by lipopolysaccharides [13]. In other studies, 0.1% DMSO altered the epigenetic landscape of cardiac and hepatic cells [17] or induced differentiation in embryonic cells [7] with consequences on the results obtained from the cell assays. The other widely used solvent EtOH was also found to induce embryonic cell differentiation at a concentration of 0.25%, which indicates that the experimental design should include, together with the selection of a suitable solvent, an assessment of its acceptable concentrations for the selected assay. The PEG derivatives are often preferred for solubilization of commonly less soluble compounds. Some low molecular weight PEG polymers (PEG-200) were found to be clastogenic in a Chinese hamster liver cell line at concentrations of 5–7 mM [18]. Higher molecular weight PEG derivatives exhibited cytotoxicity in concentrations up to 20 mg/mL on HeLa and L929 (CCL-1) cells [12] and were found to be more suitable for dissolution of substances for in vitro experiments with cell lines. The solvent DMF was found to be more cytotoxic than DMSO and EtOH in studies on breast cancer, mouse macrophage and human umbilical vein endothelial cells, showing a maximum tolerated concentration (MTC) of 0.1%, whereas DMSO, EtOH and acetone could be applied in concentrations up to 0.5% *v/v* [9]. The effects of DME and MEtOH were not studied on cell lines.

Concerning the sensitivity of microorganisms, a diverse solvent tolerance on the growth of different bacterial species was observed for MeOH, EtOH, DMF and DMSO. *Escherichia coli* and *Bacillus subtilis* were able to grow on agar plates in the presence of up to 4.8% of the solvents [11]. DMF was more toxic to *E. coli* than the other solvents. *B. subtilis* was shown to tolerated higher concentrations of DMF (≥10% *v/v*), suggesting that it can use DMF as a source of carbon and nitrogen. A study performed with DMSO, EtOH and MeOH on *Staphylococcus epidermidis*, *Salmonella paratyphi* A, *Vibrio cholerae*, *Shigella flexneri* and *Pseudomonas oleovorans* found that EtOH was mostly toxic to the listed microorganisms, inhibiting their growth by 19% at a concentration of 1% *v/v*, followed by MeOH. In contrast, DMSO was the least cytotoxic, with MTC of 3% volume fraction [8].

The available literature concerning the sensitivity of animal and human cells to the selected seven organic solvents reveals that there are no comprehensive data about their in vitro cytotoxicity on the CCL-1 and HaCaT cell lines, which are commonly used in experimental chemotherapy. CCL-1 is recommended as a standard in Annex C of ISO 10993-5 [2], and HaCaT represents a model for skin toxicity or inflammatory reactions of the skin [19]. The sensitivity to the investigated organic solvents of both cell lines was compared to that of the tumorigenic cell lines A-375 and A-431, originating from malignant melanoma and epidermoid skin carcinoma, respectively. The significance of selecting the right solvent and its impact on the readout parameters in cell assays were illustrated on the standardized concentrated extract from the plant *Vaccinium macrocarpon* (commercial product CystiCran^®^) containing 54.2% proanthocyanidins (PAC) as a test sample. Proanthocyanidins are widely used as components of different food additives due to various health effects associated with them [20]. Thus the CystiCran standardized extract is appropriate as a case example as well as a substance of interest for toxicity evaluation.

Seven pathogenic bacterial strains belonging to the genera *Escherichia*, *Yersinia*, *Pseudomonas*, *Streptococcus*, *Enterococcus* and *Staphylococcus* and one fungal strain (*Candida albicans*) were also investigated for their susceptibility to the solvents. Four of these strains are recommended by ISO 20776-1 to estimate antibacterial activity [3]. Some physicochemical properties of the solvents were also considered for a possible relationship to the solvents’ toxicities in the investigated cell lines and bacterial strains.

With this in mind, a systematic study on cytotoxicity and microbicidal activity of the most commonly used organic solvents was carried out using the MTT and AST tests. The results could help select appropriate solvents and their relevant concentrations, thus guiding experimental studies of in vitro effects of biologically active molecules or extracts. In addition, our study provides experimental data on the cytotoxic effects of the standardized extract from the plant *Vaccinium macrocarpon,* depending on the solvents used and cell lines tested.

## 2. Materials and Methods

### 2.1. Chemicals and Reagents

The following chemicals were purchased from Merck (Sigma-Aldrich, Steinheim, Germany): dimethyl sulfoxide (DMSO, CAS 67-68-5, #D2650), ethanol (EtOH, CAS 64–17-5, #1009831011, ACS reagent) and methanol (MeOH, CAS 67-56-1, #34860-1L-R, ≥99.9%), 3-(4,5-dimethylthiazolyl-2)-2,5-diphenyltetrazolium bromide (MTT, #M2128-1G), disodium ethylenediamine-tetraacetate dihydrate (EDTA, #E6635), L-glutamine (#G7513), and Dulbecco’s phosphate-buffered saline (PBS, #D8537). N,N-dimethylformamide (DMF, CAS 68-12-2, ≥99.8%, ACS reagent), polyethylene glycol of average molecular weight 400 (PEG-400, CAS 25322-68-3); 2-methoxyethanol (MEtOH, CAS 109–86–4, 99.5+%, for analysis) and 1,2-dimethoxyethane (DME, CAS 110-71-4, 99+%, extra pure, SLR) originated from Acros Organics (Thermo Fisher Scientific, Waltham, MA, USA). The media, enzymes and sera used for the in vitro experiments with human cell lines originated from Capricorn Scientific, Ebsdorfergrund, Germany: DMEM (#DMEM-HPA), MEM (#MEM-A), fetal bovine serum (#FBS-HI-12A), horse serum (#HOS-1A), pen/strep 100*x* (#PS-B), trypsin (#TRY-1B10, # TRY-2B10), and Accutase^®^ (#ACC-1B). HCOOH was from Chimspektar OOD, Sofia, Bulgaria. The following media were used for the in vitro experiments with bacteria: brain heart infusion broth/agar (#M210/#M211, HiMedia, Mumbai, India), Mueller–Hinton broth/agar (#CM0405B, #CM0337B, Thermo Scientific—Oxoid, Basingstoke, UK) and Luria–Bertani (LB) broth/agar (#CM0996B, Thermo Scientific—Oxoid, Basingstoke, UK). CystiCran^®^, a standardized concentrated extract from *Vaccinium macrocarpon* with 54.2% content of PAC A (against minimum 40% proanthocyanidins, according to the Food, Drug and Cosmetic Act requirements, without preservatives, flavoring or coloring), was delivered by NATUREX-DBS, LLC (Sagamore, MA, USA).

### 2.2. Cell Lines and Culture Conditions

The cell lines utilized in this study included the human tumor lines A-375 (malignant melanoma) and A-431 (non-melanoma epidermoid squamous skin carcinoma) as well as the models for non-tumorigenic cells—HaCaT (immortalized human keratinocytes) and CCL-1^TM^ (transformed mouse fibroblasts, NCTC clone 929). The first three cell lines were obtained from CLS Cell Lines Service (GmbH, Eppelheim, Germany). CCL-1^TM^ was purchased from the American Type Culture Collection (ATCC, Manassas, VA, USA). Cells were maintained in a controlled environment—sterile cell culture flasks in a CO_2_ incubator Panasonic MCO-18AC (Panasonic Healthcare co., ltd, Oizumi-Machi, Japan) at 37 °C and a humidified atmosphere containing 5% CO_2_. CCL-1 was cultured in MEM with 10% heat-inactivated horse serum and 2 mM L-glutamine, and the cell culture medium for the rest of the cell lines was DMEM-HG with 4.5 g/L glucose with the addition of 10% heat-inactivated fetal bovine serum and 4 mM L-glutamine. Pen/strep (concentration of penicillin G sodium 10^5^ Units/L and of streptomycin sulfate 100 mg/L) was added to both culture media. The cell lines were maintained as an adherent monolayer in a log phase. The cells were sub-cultivated about 1–3 times per week after the monolayer reached 80–90% confluence. The medium was removed from the flask, and the cell monolayer was washed with 3–5 mL PBS. The cells were incubated with 0.05% EDTA in PBS for 8–10 min, followed by the next step where all cell lines were subjected to enzyme cell detachment. HaCaT and CCL-1 monolayers were detached with 1 mL solution of trypsin/EDTA (0.1%/0.05% for HaCaT and 0.25% (*w/v*)/0.53 mM for CCL-1) and incubated for 2–5 min at 37 °C (for HaCaT) or at room temperature (for CCL-1 cells), whereas Accutase^®^ was used for A-375 and A-431 lines. The incubation lasted until the separation and dispersion of the cells in the monolayer were observed using light microscopy. In the end, all cell lines were resuspended in 10 mL medium and centrifuged for 3 min at 300× g. The cell pellet was resuspended in fresh medium and sub-cultivated at a seeding density of 1.0 × 10^4^ cells/cm^2^.

### 2.3. Bacterial and Fungal Strains and Culture Conditions

The bacterial strains used for AST of the solvents included: *Staphylococcus aureus* (ATCC 29213), *Staphylococcus aureus*—MRSA (NBIMCC 8327—resistant to methicillin and oxacillin, National Bulgarian Collection for Industrial Microorganisms and Cell Cultures, Sofia, Bulgaria), *Streptococcus pyogenes* (SAIMC 10535, Collection of the Stephan Angeloff Institute of Microbiology, Sofia, Bulgaria), *Enterococcus faecalis* (ATCC 29212), *Escherichia coli* (ATCC 35218), *Pseudomonas aeruginosa* (ATCC 27853), and *Yersinia enterocolitica* (IP864 O:3, Collection of Institut Pasteur, Paris, France). The selected strains are the most often investigated ones in the AST research studies in correspondence with the standard ISO 20776-1 Part 1: Reference method for testing the in vitro activity of antimicrobial agents against rapidly growing aerobic bacteria involved in infectious diseases [3]. The antimycotic activity was tested on *Candida albicans* (CBS 562, Utrecht, The Netherlands). The strains *S. aureus*, MRSA, *E. coli* and *E. faecalis* were grown on Mueller–Hinton broth/agar (MHB/MHA); *S. pyogenes*, *P. aeruginosa*, *Y. enterocolitica* and *C. albicans* were grown on brain heart infusion (BHI) broth/agar; *E. coli* was also grown on Luria–Bertani broth/agar (LBB/LBA).

### 2.4. Cytotoxicity Assessment (MTT Dye-Reduction Assay) on Cell Lines

The MTT test was performed in order to determine the median inhibitory (IC_50_) and MTC concentrations of the solvents and PAC according to Annex C, ISO 10993-5 [2,21]. Briefly, 100 µL of the cell suspension (1 × 10^5^ cells/mL) were seeded per well in 96-well plates with a flat bottom. The cells were incubated for 24 h, allowing them to enter the log phase of their growth. On the 24th hour, the cells were treated with the solvents or CystiCran^®^ solutions in concentrations ranging between 0.004% and 2% volume fraction (*v/v*) and incubated further for 72 h. The incubation periods are following the recommendations of the FDA guide [22] for the use of ISO 10993-1 [1]. At the end of the incubation period, 10 µL of an MTT solution (5 mg/mL) were added to each well, and the plates were kept at 37 °C for 2 hours. Thereafter, the medium was aspirated, and the formed formazan crystals were dissolved in 100 μL/well of 2-propanol supplemented with 5% formic acid. The same solvent was used as a blank. Untreated cells were considered as a negative control. The highest concentration of DMSO applied (2% volume fraction) served as a positive control. The absorption was measured at 540 nm (reference filter at 690 nm) on a microplate reader ELx800 (BioTek Instruments, Inc., Winooski, VT, Winooski, VT, USA).

### 2.5. Determination of Minimal Inhibitory and Bactericidal Concentrations

The minimum inhibitory concentration (MIC) for each solvent was estimated by the broth microdilution method (BMD) in 96-well microplates, following ISO 20776/1-2006 [3] and CLSI protocols [23]. Briefly, a standardized bacterial inoculum was prepared from each bacterial species by suspending 3–4 colonies from a pure culture into 4 mL sterile saline. The turbidity of the inocula was adjusted to McFarland 0.5 (1.0 × 10^8^ CFU/mL, λ = 600 nm), and 50 µL were resuspended in 10 mL of the appropriate culture medium for each strain. The bacterial inocula were added to microtiter trays containing equivalent volumes of medium loaded with the relevant solvent in concentrations varying from 0.025 to 25% volume fraction. The final volume of each sample was 100 µL/well. The plates were incubated at 37 °C for 24 h. The pure culture medium served as a negative control. Every MIC was evaluated visually as the lowest concentration without visible growth. The minimum bactericidal concentration (MBC) was determined by incubating the samples treated from ½ MIC up to the highest concentration on MHA for 24 h at 37 °C. MBC was defined as the lowest compound’s concentration reducing the colony growth of the initial bacterial inoculum by ≥ 99.9%.

### 2.6. Dehydrogenase Activity Test

The cell dehydrogenase (DEHA, redox, metabolic) activity of all samples was measured at the end of the BMD assay, based on reducing the MTT dye to formazan by the membrane located bacterial enzyme NADH: ubiquinone reductase (H^+^-translocation). The protocol of Wang et al. was applied after minor modifications [24]. Briefly, each sample was incubated for 120 min at 37 °C with MTT at a final concentration of 0.5 mg/mL in the culture medium. The resulting non-soluble violet formazan crystals were dissolved with an equal volume of 2-propanol supplemented with 5% formic acid. The absorbance was measured at 550 nm against a blank solution containing the respective volumes of MHB and MTT.

### 2.7. UV-Spectrophotometric Method

The stock solutions of CystiCran^®^ were prepared by dissolving and mixing adequate amounts of the substance in water, EtOH, DMSO or MEtOH to obtain a concentration of 5 mg/mL. Thereafter, 1.0 mL of each stock solution was diluted in 10.0 mL of the respective solvent to a concentration of 0.5 mg/mL. A volume of 0.1 mL of each 0.5 mg/mL solution was further diluted in 25.0 mL of the respective solvent to a final concentration of 0.002 mg/mL. These solutions were subjected to spectrophotometric analysis (UV-vis spectrometer HP) against the respective blanks—water, EtOH, DMSO and MEtOH. The analytical calculations were based on multicomponent analysis calculations, derivative order 0, polynomial degree 0, smoothing points 1, data interval 2 nm and analytical wavelength zone from 190 nm to 820 nm.

### 2.8. Statistical Analysis

The IC_50_ values from the MTT assay were evaluated with a non-linear regression analysis (Curve fit, GraphPad Prizm 6.01 software, GraphPad Software Inc., San Diego, CA, USA) using sigmoidal concentration–response curves. The cell viability is presented as a percentage of the untreated control. At least four wells were seeded for every concentration. Three independent assays were performed (*n* = 3). The maximum tolerated concentration (MTC) for the cell lines was determined from three independent assays according to ISO 10993-5 as the maximum concentration at which at least 70% of the cells were viable (nonlin fit, range, GraphPad Prizm 6.01 software) GraphPad Software Inc., San Diego, CA, USA). To compare the MTC values of the solvents for each cell line, the ordinary One-way ANOVA statistical analysis based on Tukey’s multiple comparisons test with a single pooled variance (GraphPad Prizm 6.01 software) GraphPad Software Inc., San Diego, CA, USA) was utilized, and *p* ≤ 0.05 was set as a significance level. The respiratory activity of the bacterial cultures was calculated as a percentage against the untreated control.

### 2.9. Calculation of Solvents’ Physicochemical Properties

Molecular property calculations were performed on the Percepta Platform of ACD/Labs, v. 2020.2 (Advanced Chemistry Development, Inc., Toronto, On, Canada, www.acdlabs.com) and ChemAxon’s Chemicalize platform (April 2021, https://chemicalize.com/, accessed on 31 March 2021). The following physicochemical properties were calculated: the octanol-water partition coefficient (logP), the distribution coefficient (logD) at pH = 7.4, and the topological polar surface area (TPSA). TPSA calculation is based on the summation of tabulated surface contributions of polar fragments in the chemical structures, and it is conformationally independent [25].

## 3. Results

### 3.1. In Vitro Cytotoxicity of Solvents on Tumorigenic and Non-Tumorigenic Cell Lines

**Figure 1:**
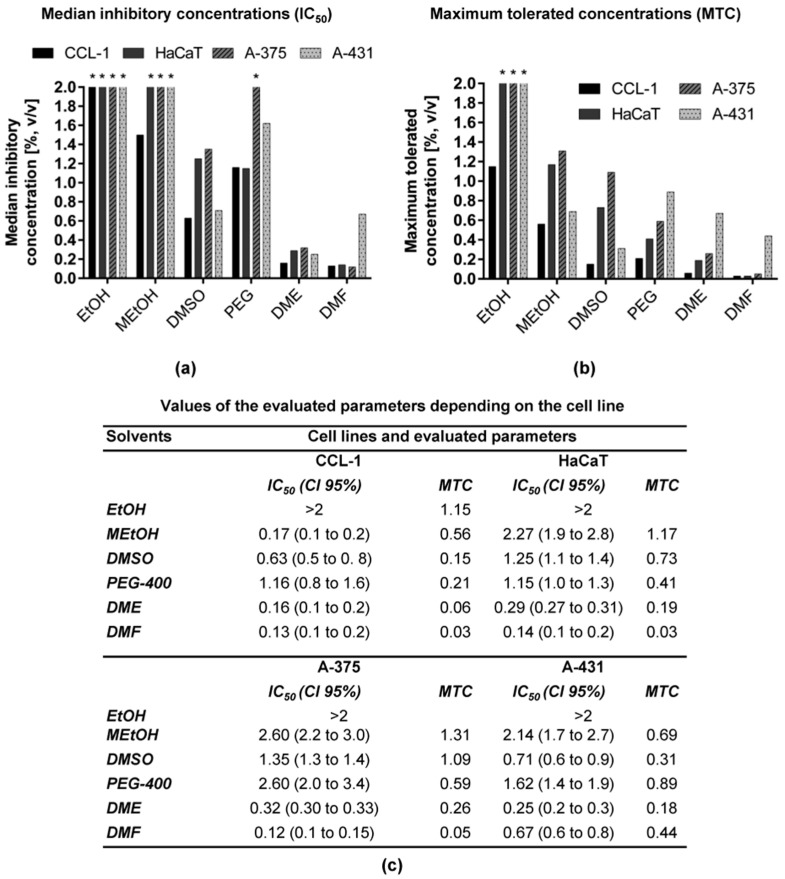
(**a**) IC_50_ and (**b**) MTC of six solvents expressed as % (*v/v*) of solvents on a panel of four cell lines after 72 h incubation; (**c**) numerical values of IC_50_ and MTC based on three independent assays (*n* = 3) and used in (**a**,**b**), respectively. Legend: IC_50_—median inhibitory concentration; MTC—maximum tolerated concentration; CI 95%—confidence interval 95%. Asterisks (*) denote that the IC_50_ or MTC values exceed the maximum tested concentration.

**Figure 2:**
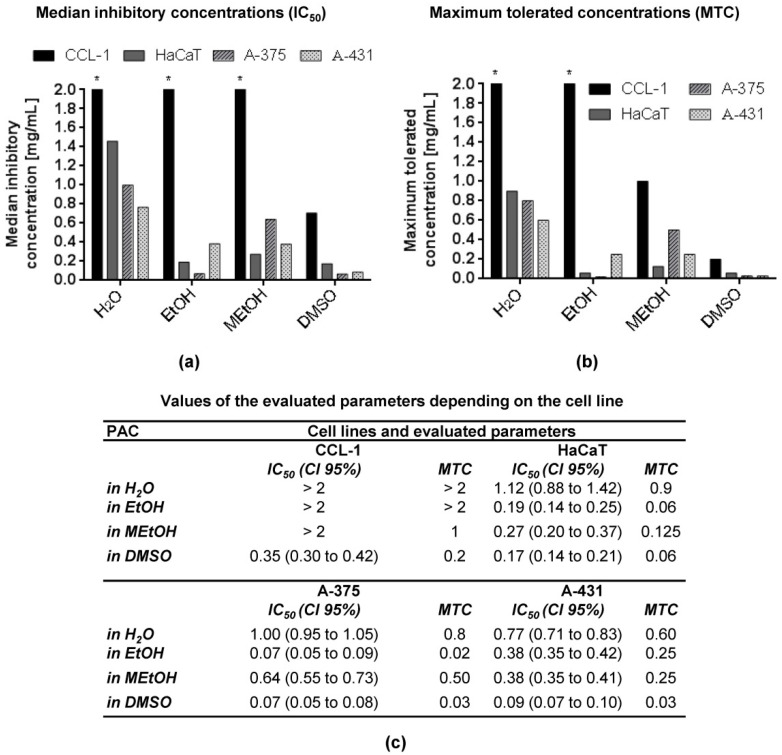
(**a**) IC_50_ and (**b**) MTC of CystiCran^®^ dissolved in four different solvents on a panel of four cell lines after 72 h of incubation. (**c**) Numerical values of IC_50_ and MTC based on three independent assays (*n* = 3) and used in (**a**,**b**), respectively. Legend: IC_50_—median inhibitory concentration; MTC—maximum tolerated concentration; CI 95%—confidence interval 95%. Asterisks (*) denote that the IC_50_ or MTC values exceed the maximum tested concentration.

The results from the evaluation of the in vitro cytotoxicity of six common organic solvents and PAC (dissolved in the least toxic three of them at a concentration of 50 mg/mL) on four cell lines with the MTT test are summarized in Figure 1 and Figure 2.

Figure 1a,b illustrates a graphical comparison of the four cell lines regarding the IC_50_ values and MTC of the solvents, respectively, whereas the values are presented as numbers in Figure 1c. The IC_50_ of the solvents varied from 0.12 to >2% *v/v* and the MTCs were in the range of 0.03 to >2% *v/v* (Figure 1). The IC_50_ values of EtOH were not calculated, as this solvent did not influence the proliferation of the four tested cell lines up to a concentration of 2% volume fraction, except for CCL-1. The MTC of EtOH leading to less than 30% inhibition of this cell line proliferation was 1.15% *v/v*. The IC_50_ values greater than 2% *v/v* were calculated for MEtOH on HaCaT, A-375 and A-431 cells and for PEG-400 on A-375 cells. The solvents showing IC_50_ values between 1 and 2% *v/v* were DMSO on HaCaT and A-375 and PEG-400 on CCL-1, HaCaT and A-435. DME and DMF showed IC_50_ values lower than 1% *v/v* for all four examined cell lines. The MTC values over 2% *v/v* were measured only for EtOH on HaCaT, A-375 and A-431. MEtOH was not cytotoxic in concentrations higher than 1% *v/v* for HaCaT and A-375 cell lines, whereas 1% DMSO was tolerable only for A-375 cells. All other solvents were tolerable in concentrations between 0.03% *v/v* (DMF on CCL-1 and HaCaT) and 0.89% *v/v* (PEG-400 on A-431). As expected, EtOH was the least cytotoxic solvent, with MTC and IC_50_ > 2% *v/v* determined on HaCaT, A-375 and A-431 cell lines. It was followed by MEtOH with MTCs ranging from 0.56 to 1.31% and IC_50_ 0.17 to 2.60%. Next were DMSO and PEG-400, which exhibited similar toxicity—MTCs 0.15 to 1.09% and IC_50_ 0.63 to 2.60%. The most toxic solvents were DME and DMF—their MTCs were in the range of 0.03–0.67% and IC_50_ varied in the interval 0.12–0.67%.

### 3.2. In Vitro Cytotoxicity of CystiCran^®^ on Tumorigenic and Non-Tumorigenic Cell Lines

The results for the in vitro cytotoxicity of the concentrated PAC extract CystiCran^®^ are presented in Figure 2.

All four cell lines (CCL-1, HaCaT, A-375 and A-431) were incubated with different concentrations of the extract (0.004–1 mg/mL) for 72 h. The IC_50_ values for CCL-1 when the PAC extracts were dissolved in water, EtOH or MEtOH were not calculated because the values were higher than the highest concentration applied. The remaining IC_50_ values varied between 0.07 and 1.12 mg/mL. The MTC ranged between 0.02 and 1 mg/mL. The lowest IC_50_ value was that of CystiCran^®^ in DMSO or EtOH on A-375 cells, and the highest (except CCL-1 cells) was determined for HaCaT cells when water was used as a solvent. The least sensitive cell line was CCL-1, and the most sensitive—A-375. The DMSO solution was less cytotoxic for HaCaT (IC_50_ = 0.17 mg/mL) than for A-431 (IC_50_ = 0.09 mg/mL) and A-375 (IC_50_ = 0.07 mg/mL) cells. The EtOH solution was the least cytotoxic to CCL-1 cells, more cytotoxic to A-431 (IC_50_ = 0.38 mg/mL) and HaCaT (IC_50_ = 0.19 mg/mL) and the most cytotoxic—to A-375 (IC_50_ = 0.07 mg/mL) cells. The IC_50_ values for MEtOH were between 0.27 and 0.64 mg/mL, whereby HaCaT was the most sensitive, followed by A-431 and A-375.

### 3.3. Spectrophotometric Evaluation of the Absorbance of CystiCran^®^ in Different Solvents

Based on the above-described cytotoxicity studies, spectrophotometric measurements were performed using EtOH, DMSO and MEtOH. The solvents DME, DMF and PEG-400 were not tested due to their high cytotoxicity. The results obtained in the course of the study in the analytical zone of 190–820 nm wavelength (λ) showed the presence of several absorption maxima (λ_max_) as follows: 1) water—216, 242, 280 and 538 nm without an isolated peak at 366 nm; 2) absolute EtOH—242, 280, 366 and 538 nm; 3) DMSO—280, 366 and 538 nm; 4) MEtOH—242, 280, 366 and 538 nm. The absorbance values at λ_max_ 242, 280 and 366 nm in the different solvents are presented in Table 1.

### 3.4. Susceptibility of Bacterial Strains

Table 2 reports on the MIC, MBC and DEHA activity of the tested bacterial strains towards the six solvents commonly used in the AST. DME was the most toxic, with MIC ranging between 3.125 (for MRSA, *Y. enterocolitica* and *E. coli* in LBB) and 12.5% *v/v* (for *E. faecalis, E. coli in MHB* and *P. aeruginosa*), followed by DMSO with MICs between 3.125 (for *Y. enterocolitica*) and 25% *v/v* (for the *Staphylococcus aureus* strains). The MICs determined for MEtOH were from 6.25 to 25% *v/v* for *P. aeruginosa* and the staphylococcal strains, respectively. The least toxic were EtOH and MEtOH, with MICs higher than 25% *v/v* for all tested bacterial strains. The solvents EtOH and MEtOH did not also inhibit the respiratory activity of the treated bacteria, whereas the other four solvents showed a varying inhibition potential on the tested strains. There was no proportional dose-dependent correlation between the MIC values and the DEHA activity. The MBC was higher than the MIC for most of the strains and solvents, with some exceptions (DME and MEtOH in *S. aureus*, DME in *E. faecalis*, DME for *E. coli* in MHB, MEtOH for *E. coli* in LBB, DME for *P. aeruginosa* and PEG-400 for *Y. enterocolitica*).

### 3.5. Susceptibility of the Fungal Strain Candida Albicans

The growth of *C. albicans* was inhibited at a concentration of 6.25% *v/v* MEtOH or DMSO (Table 3). Regarding DME, 12.5% were needed to achieve the same effect. PEG-400, EtOH and MeOH were not toxic at the highest concentrations tested—25% *v/v*. Only MEtOH and DME reduced the metabolic activity of the strain, with DME being a stronger inhibitor (31.23% active cells) than MEtOH (>70% active cells). The MBCs were higher than the MICs, except for DMSO, which exhibited bactericidal effect at the same concentration determined as MIC—6.25% *v/v*.

### 3.6. Physicochemical Parameters of the Studied Solvents

Table 4 reports on the solvents’ experimental and calculated values of some of the most important physicochemical parameters used to characterize the pharmacokinetic behavior of the bioactive compounds: logP, logD at pH = 7.4, and TPSA. There was a difference in the logP, and logD calculations between the software platforms ACD/Labs and ChemAxon used in the calculations. Thus the average of both values was reported for each solvent. Equivalent TPSA values were produced by both software programs as they use the same methodology for the calculation of TPSA [25].

## 4. Discussion

There are few research models on cytotoxicity of vehicle solvents by using transformed non-tumorigenic cell lines [9,12,24]. In our study, we have implemented for the first time such experiments on HaCaT and CCL-1 cells, except for PEG-400 [12], which was already tested on CCL-1, but for different incubation periods (24 h). In addition, there is no information in the available literature about the AST of *Yersinia* strains regarding all studied solvents or the effects of DME, MEtOH and PEG-400 on the bacterial test strains presented here. To determine better the applicability of the tested solvents and their maximum nontoxic concentrations, we applied bioassays recommended for evaluation on in vitro cytotoxicity in cell lines and AST of microorganisms by international standard protocols or widely used laboratory assays [1,2,3,21,24]. The described protocols are a reliable basis for initial high-throughput screening of extracts and compounds for biological activities, aiming to select the most active ones. The evaluated parameters for the cell lines included IC_50_ values and MTCs, whereas MICs, MBCs and respiratory activity were measured for the bacterial cultures.

Generally, the non-tumorigenic cell lines tested in our study were more sensitive than the tumorigenic ones. The mouse fibroblasts were more sensitive than the immortalized keratinocytes, as seen from the lower IC_50_ and MTCs values (Figure 1) of all solvents, except for PEG-400, which inhibited the proliferation of both cell lines at the same concentration (1.15–1.16% *v/v*). However, the MTC of PEG-400 in HaCaT (0.41% *v/v*) was twofold higher than that in CCL-1 (0.21% *v/v*), confirming the general trend of the higher sensitivity of fibroblasts. A plausible explanation for these results is the function and distribution of both cell types in human or animal tissues. Keratinocytes are present in all four epidermal layers, and they function as a protective barrier against adverse environmental factors [27,28], whereas fibroblasts are mesenchymal cells, which are not directly exposed to the environment [29,30]. Our results are in line with the data published in a comparative study on the sensitivity of primary and transformed keratinocytes and fibroblasts against skin-irritating substances where mouse fibroblasts (3T3) were also found to be more sensitive to DMSO and propylene glycol than HaCaT cells or multilayer culture of primary keratinocytes [31].

In our study, EtOH was among the solvents with the weakest cytotoxicity towards mouse carcinoma cells, in agreement with a study performed by [5]. Similarly, EtOH exerted the least cytotoxic effect, and DMSO exhibited much less cytotoxicity than other solvents towards non-tumorigenic cell lines (mouse and human again) in a previous study [14]. According to Singh [10], DMSO exhibits no cytotoxicity on skin fibroblast cells at 0.1% DMSO, but the higher concentrations (0.5–3%) lead to a reduction in cell viability in a dose-dependent manner, with a significant cytotoxic effect, starting from 1%. In our study, DMSO inhibited less than 30% proliferation of skin keratinocytes at 0.73% *v/v*, which was determined as MTC for this solvent in this cell line, and the concentration of 1.25% *v/v* led to 50% inhibition, which is consistent with the results of [10]. In another research involving human and mouse tumor and normal human cell lines, EtOH and DMSO at concentrations of 0.1% and 0.5% had little or no toxicity, whereas higher concentrations inhibited the cells’ growth again DMF displayed higher toxicity in agreement with [9]. Studies conducted to assess the toxicity of PEG-400 have shown that it depends on the respective derivative and the cell line [12,18]. The PEG-400 used in our study exerted an IC_50_ value of 13 mg/mL (1.16% *v/v*) for CCL-1 cells, which is lower than the cytotoxicity estimated by [12], due to, most probably, the difference in the incubation time—72 h in our experiment vs. 24 h in the other study.

The impact of the three least toxic solvents on the cytotoxicity of the plant product CystiCran^®^, containing a high concentration of PAC, was tested on four cell lines. PACs, the oligomers and polymers of flavan 3-ols, belong to the flavonoid family. PACs found in cranberry (*Vaccinium macrocarpon*) appear to be of primary importance for preventing uropathogenic bacterial adhesion [32,33,34,35]. As cranberry extracts are constantly being studied to characterize their biological effects and cytotoxicity, we focused on CystiCran^®^ as a suitable test product to assess the importance of different solvents in the planning and conducting of bioassays. As seen in Figure 2, there were significant differences in the cytotoxic effect of CystiCran^®^ when dissolved in different solvents. The trend showed a dependence on the cytotoxicity of the solvents themselves. Namely, the substance was more cytotoxic when dissolved in a more cytotoxic solvent, such as DMSO. This dependence varied among the different cell lines, probably due to the cell type. The sensitivity of the non-tumorigenic cells to the water solution of CystiCran^®^ was significantly lower (IC_50_ = 1.12 mg/mL for HaCaT and > 2 for CCL-1) than that of the tumor cell lines (IC_50_ = 1 for A-375 and 0.77 for A-431), which was not the case for EtOH and MEtOH. To investigate if the observed differences are due to changes in the physicochemical features of the solutions, we measured their absorption spectrophotometrically. UV spectroscopy is considered to be one of the methods in the structural analysis of PAC: the compounds absorb principally in the range of 240 to 400 nm, and the UV spectra of most of them are composed of main absorption maxima between 300 and 400 nm and 240–285 nm. During the studies, changes were reported in both analytical zones. For all solutions except water, the absorption maxima disappeared at 216 nm, and for the solution in DMSO—at 242 nm. Again at 242 nm, the absorbance values for the other solutions decreased, and a hypochromic effect was observed. Only for MetOH the absorbance values at 280 and 366 nm were higher (hyperchromic effect). At 538 nm, the absorbance values of all solutions were below 0.190 AU (absorption units). All spectral profiles reflected changes in the structure and composition of the CystiCran^®^ components. The polar solvents used have a negligible effect on λ_max_ regardless of their interaction with the PAC molecules. Hypochromic and hyperchromic effects only are available compared to water, which mainly influences the quantitative content of PAC. A comparison between the IC_50_ values of the solvents (Figure 1) and CystiCran^®^ (Figure 2) led to the assumption that the in vitro cytotoxicity of the extract was mainly affected by the cytotoxicity of the relevant solvents.

Regarding the bacterial strains, EtOH, MeOH and PEG-400 proved to be least cytotoxic, which makes them attractive solvents for plant extracts and other substances in AST experiments. As seen in Table 2, neither EtOH nor MeOH influence the respiratory activity of the tested strains when concentrations below 25% *v/v* are applied. This assay is related to the enzymes involved in ATP production, which points to the suggestion that this concentration would not interfere with signal transduction and quorum sensing experiments. Unlike them, PEG-400 suppressed the respiratory activity of *E. coli* and *P. aeruginosa* by approximately 76% and 85%, respectively, thus defining the *P. aeruginosa* strain as more sensitive than the *E. coli* one. The results obtained for EtOH and MeOH are partially consistent with other published data about both solvents and their effects on *E. coli* [8,11]. In our study, MeOH and EtOH were found to be less toxic than DMSO. The MBCs determined herewith were higher than 25% *v/v*, and the bacterial growth was not inhibited at this concentration, unlike the report of Dyrda et al. [11]. In our study, we noted that the bacterial culture medium might also influence the toxicity of the solvents. To prove that, we compared the inhibitory and bactericidal effects of all solvents on *E. coli* cultured in MHB and LBB. The latter is less rich in nutrition components than MHB, and, as expected, bacteria cultured in LBB were more sensitive to the solvents than those maintained in MHB (Table 2). Wadhwani et al. tested DMSO, MeOH and EtOH in concentrations from 1 to 6% *v/v* and reported lower doses inhibiting cell growth than the doses determined in our research [8]. The sensitivity of the *Pseudomonas* strains probably varies due to two reasons—specificity of the species (the authors used *P. oleovorans*, whereas the strain in our panel was *P. aeruginosa*) and difference in the culture media. For the BMD test, we cultured the *P. aeruginosa* strain in BHI broth, which is richer in nutrition components than the MHB used in [8]. The same difference was observed in the results for the strains from the genera *Staphylococcus*. The *S. epidermidis* strain investigated by Wadhwani et al. [8] was more sensitive to the three solvents (26% survival in the presence of 6% MeOH or EtOH and only 15%—in the presence of DMSO) than both *S. aureus* strains tested in our experiments. Only DMSO in concentration 25% *v/v* inhibited the metabolism of both strains up to 12–13% metabolically active cells, but did not exert any bactericidal effect, showing full restoration of the bacterial growth 48 h later and after culturing on agar plates. Interestingly, the *Y. enterocolitica* strain was the most sensitive to the solvents tested, compared to the other strains. Its growth was inhibited at 3.125% *v/v* DMSO or DME, and at 12.5% *v/v* PEG-400 or MEtOH. Only EtOH and MeOH were well tolerated, exerting no toxicity on this strain at concentrations of 25% *v/v* or higher.

The analysis of data on *C. albicans* showed that the fungal cells were the most sensitive to DMSO, followed by MEtOH and DME, which made them not suitable for further evaluation of antifungal activity on this strain. Moreover, MEtOH and DME suppressed the metabolic activity of this strain and could interfere with experiments on enzymatic activity. The other three solvents, PEG-400, EtOH and MeOH, were tolerable in concentrations up to 25% volume fraction.

A comparative analysis of the cytotoxicity demonstrated by various solvents depending on the cell line and bacterial/fungal strain was performed by following the IC_50_, MTC, MIC, MBC and DEHA values. In the summary presented in Table 5, the solvents were characterized by the level of toxicity for each strain. In general, considering the results obtained, the most toxic solvents in our study were DMF and DME, and they could not be recommended for use on the tested cell lines. Regarding the other solvents, EtOH is the most suitable for application as it is appropriate for the dissolution of a broader range of extracts and compounds, depending on their polarity. For strongly nonpolar test materials, MEtOH or PEG-400 could be the solvent of choice, depending on the cell line—MEtOH for CCL-1, HaCaT and A-375, and PEG-400 for A-431 cells. DMSO could be applied for the dissolution of substances to be tested on A-375 cells if there is no other possibility. As DMSO induces epigenetic changes and modulates other basic cellular processes, such as microRNA deregulations and the expression of more than 2000 genes at a concentration of 0.1% *v/v* [17], we could conclude that its use as a solvent for the tested cell lines would interfere with the results of cytotoxic assays and investigation of proteomic and epigenetic changes, especially in the tumorigenic cell lines if they are the object of in vitro oncopharmacological experiments in the area of the experimental chemotherapy. The most toxic for the bacteria was DME, followed by DMSO and MEtOH, which points to the suggestion that MeOH, EtOH and PEG-400 are more suitable solvents for AST assays (Table 5).

Having in mind that the studied solvents are organic compounds with their own pharmacokinetics profile, we also looked for possible relationships between some physicochemical properties of the solvents and their cytotoxicity and microbicidal activity recorded in our experiments. Comparing the reported physicochemical parameters and the ranking of the solvents (Table 4 and Table 5), no clear dependence of toxicity on the physicochemical properties of the studied solvents could be outlined. This suggests that the solvents’ in vitro effects are complex and certainly depend on a variety of factors, including not only the physicochemical nature of the solvent but also the type of the cell line or the pathogenic microbial species under investigation. This is in agreement with the study of [11], where it was shown that the toxicity of the solvent strongly depends on the bacterial species and may completely inhibit the growth of one organism while being more or less tolerated by another one. These results point to the necessity of conducting more experimental studies using different methods, like, for example, scanning and transmission electron microscopy, to get a deeper insight into the mechanisms of tolerance and susceptibility of the cells to the studied organic solvents. In addition, more substances must be subjected to such a systematic analysis to extend the spectrum of the case studies. Such investigations are currently in progress.

## 5. Conclusions

To the best of our knowledge, the presented study is among the very few ones that investigate systematically the cytotoxic and microbicidal effects of the most used organic solvents and compares their effects on a panel of non-tumorigenic and malignant cells, bacteria and fungi. The non-tumorigenic cells prove to be more sensitive than the tumorigenic ones to all studied solvents. EtOH, MEtOH and PEG-400 are better tolerated by the cell lines in concentrations between 1 and 2% *v/v* than DMSO, DME and DMF. The most sensitive cell line is CCL-1. The cytotoxicity of the solvents influenced the cytotoxic effects of the extract CystiCran^®^, which confirms the need to predetermine the most suitable solvent when planning bioassays to evaluate in vitro cytotoxicity and to develop the experimental design. Additionally, EtOH and MeOH do not affect bacterial and fungal growth in a concentration up to 25%. PEG-400 and MEtOH are moderately tolerated by bacteria, but the latter has more pronounced antifungal activity. DME is the most toxic to all tested bacterial species, except for *S. pyogenes* and *C. albicans* (≥25%). DMSO shows microbicidal effect in concentration 6.25% only against *Y. enterocolitica* and *C. albicans*. The presented study facilitates the appropriate solvent selection that is extremely important when evaluating the effects of biologically active molecules or extracts in MTT and AST assays. It can help guide researchers to decide on the most relevant solvent and concentrations to be used in in vitro experiments that deal with the herewith examined cell lines and bacterial strains.

## Figures and Tables

**Table 1:** Absorption values (A) of CystiCran^®^ solutions at λ_max_ of 242, 280 and 366 nm in different solvents.

Solvent	A_1_ at 242 nm	A_2_ at 280 nm	A_3_ at 366 nm
Water	3.092	1.985	0.585
EtOH	2.920	1.994	0.660
DMSO	−	1.762	0.487
MEtOH	2.461	2.552	0.838

**Table 2:** Susceptibility of seven bacterial strains to six commonly used organic solvents after 24 h of exposure.

Solvents	Bacterial Strains and Evaluated Parameters					
	*S. Aureus* ATCC 29213			*S. Aureus* MRSA NBIMCC 8327		
	MIC (%)	DEHA (% ± SD)	MBC (%)	MIC (%)	DEHA (% ± SD)	MBC (%)
MEtOH	25	10.27 ± 0.01	>25	25	–	25
DME	6.25	12.54 ± 0.03	12.5	3.125	–	3.125
PEG-400	>25	–	>25	25	–	25
EtOH	>25	–	>25	>25	–	>25
DMSO	25	12.29 ± 0.04	>25	25	13.01 ± 0.05	>25
MeOH	>25	–	>25	>25	–	>25
	***S. pyogenes* SAIMC 10535**			***E. faecalis* ATCC 29212**		
	**MIC (%)**	**DEHA** **(% ± SD)**	**MBC (%)**	**MIC (%)**	**DEHA** **(% ± SD)**	**MBC (%)**
MEtOH	12.5	28.02 ± 0.02	>25	12.5	25.60 ± 0.04	>25
DME	6.25	31.32 ± 0.05	>25	12.5	–	12.5
PEG-400	25	23.66 ± 0.02	>25	25	65.33 ± 0.06	>25
EtOH	>25	–	>25	>25	–	>25
DMSO	12.5	27.27 ± 0.02	>25	12.5	29.86 ± 0.08	>25
MeOH	>25	–	>25	>25	–	>25
	***E. coli* ATCC 35218 in MHB**			***E. coli* ATCC 35218 in LBB**		
	**MIC (%)**	**DEHA** **(% ± SD)**	**MBC (%)**	**MIC (%)**	**DEHA** **(% ± SD)**	**MBC (%)**
MEtOH	12.5	5.86 ± 0.005	25	12.5	–	12.5
DME	12.5	–	12.5	3.125	n.m.	6.25
PEG-400	>25	–	>25	25	n.m.	>25
EtOH	>25	–	>25	>25	–	>25
DMSO	12.5	13.02	>25	12.5	n.m.	>25
MeOH	>25	–	>25	>25	–	>25
	***P. aeruginosa* ATCC 27853**			***Y. enterocolitica* IP 864 O: 3**		
	**MIC (%)**	**DEHA** **(% ± SD)**	**MBC (%)**	**MIC (%)**	**DEHA** **(% ± SD)**	**MBC (%)**
MEtOH	6.25	20.23 ± 0.06	25	12.5	11.76 ± 0.004	25
DME	12.5	–	12.5	3.125	49.04 ± 0.005	6.25
PEG-400	25	15.31 ± 0.04	>25	12.5	–	12.5
EtOH	>25	–	>25	>25	–	>25
DMSO	6.25	9.78 ± 0.01	>25	3.125	19.13 ± 0.17	6.25
MeOH	>25	–	>25	>25	–	>25

Legend: MIC—minimum inhibitory concentration; MBC—minimum bactericidal concentration; DEHA—dehydrogenase activity; LBB—Luria–Bertani broth; MHB—Mueller–Hinton broth; n.m.—not measured. The MIC, DEHA and MBC values are obtained from three independent assays (*n* = 3).

**Table 3:** Susceptibility of the fungal strain *Candida albicans* to six commonly used organic solvents after 24 h of exposure.

Solvents	Fungal Strain and Evaluated Parameters		
	*Candida Albicans* SAIMC 562		
	MIC (%)	DEHA (% ± SD)	MBC (%)
MEtOH	6.25	77.41 ± 0.03	12.5
DME	12.5	31.23 ± 0.01	25
PEG-400	>25	–	>25
EtOH	>25	–	>25
DMSO	6.25	–	6.25
MeOH	>25	–	>25

Legend: MIC—minimum inhibitory concentration; MBC—minimum bactericidal concentration; DEHA—dehydrogenase activity. The MIC, DEHA and MBC values are obtained from three independent assays (*n* = 3).

**Table 4:** Chemical structures and physicochemical properties of the studied solvents.

EtOH	MeOH	DMF	DMSO	MEtOH	DME	PEG-400 ^a^
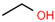	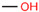	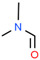	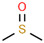	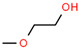	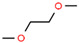	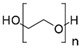
Experimental logP						
−0.31	−0.77	−1.01	−1.35	−0.77	−0.21	−4.8 [26]
Calculated logP						
−0.23 ^b^ −0.16 ^c^ −0.19 ^d^	−0.52 −0.52 −0.52	−0.78 −0.63 −0.71	−0.91 −1.41 −1.16	−0.64 −0.57 −0.61	−0.24 0.08 −0.16	− − −
Calculated logD (at pH = 7.4)						
−0.23	−0.52	−0.78	−0.91	−0.64	−0.24	−
Calculated TPSA (Å²)						
20.2	20.2	20.3	36.3	29.5	18.5	−

Legend: ^a^ The chemical formula of PEG-400 is defined as C_2n_H_4n_ + 2O_n+1_, where *n* = 8.2 to 9.1; ^b^ logP values in the row are calculated by ACD/Labs; ^c^ logP values in the row are calculated by Chem Axon; ^d^ logP values in the row are averages of the ACD/Labs and ChemAxon values.

**Table 5:** Toxicity comparison of solvents, depending on the cell line and bacterial strain.

Cell Line	Toxicity Comparison Based on MTC
CCL-1	DMF > DME > DMSO > PEG-400 > MEtOH > EtOH
HaCaT and A-375	DMF > DME > PEG-400 > DMSO > MEtOH > EtOH
A-431	DME > DMSO > DMF > MEtOH > PEG-400 > EtOH
**Bacterial strains**	**Toxicity comparison based on MIC, MBC and DEHA**
*S. aureus* ATCC 29213	DME > DMSO = MEtOH > PEG-400 = MeOH = EtOH
MRSA NBIMCC 8327	DME > MEtOH = PEG-400 > DMSO > MeOH = EtOH
*S. pyogenes* SAIMC 10535 and *E. faecalis* ATCC 29212	DME > MEtOH = DMSO > PEG-400 > MeOH = EtOH
*E. coli* ATCC 35218 in MHB or LBB	DME > MEtOH > DMSO > PEG-400 = MeOH = EtOH
*P. aeruginosa* ATCC 27853	MEtOH > DMSO > DME > PEG-400 > MeOH = EtOH
*Y. enterocolitica* IP 864	DMSO > DME > PEG-400 > MEtOH > MeOH = EtOH
**Fungal strain**	**Toxicity comparison based on MIC, MBC and DEHA**
*C. albicans* SAIMC 562	MEtOH > DMSO > DME > PEG-400 = MeOH = EtOH

## Data Availability

The data presented in this study are available on request from the corresponding author. The data are not publicly available since these data are published for the first time. The authors have no problems providing them on request.
